# Effects of forearm muscle metaboreceptors activation on sweating and cutaneous vascular responses during passive heating and cycle exercising in humans

**DOI:** 10.1186/2046-7648-4-S1-A99

**Published:** 2015-09-14

**Authors:** Tatsuro Amano, Yoshimitsu Inoue, Takeshi Nishiyasu, Glen P Kenny, Narihiko Kondo

**Affiliations:** 1 Graduate School of Human Development and Environment, Kobe University, Kobe, Japan; 2 Department of Sports Management, Osaka International University, Osaka, Japan; 3 Institute of Health and Sports Science, University of Tsukuba, Tsukuba, Japan; 4 School of Human Kinetics, University of Ottawa, Ottawa, Canada

## Introduction

Muscle metaboreceptors are afferent signals from working muscles that enhance sweating and attenuate cutaneous vasodilation during a passive heat stress in humans [1]. However, it remains unclear if metaboreceptor activation during an exercise-induced heat stress may have comparable effects on heat loss responses. This study investigates the influence of forearm muscle metaboreceptors activation on the core temperature onset thresholds and thermosensitivity (slopes) of the sweating and cutaneous vascular responses during a passive (study Ι) and exercise (study ΙΙ) induced heat stresses associated with the different modification of the responses between heating conditions [1].

## Methods

*Study Ι*: Fourteen (8 females, 6 males) young adults were passively heated for 15.5 min using a upper body water perfused suit (34 °C) and immersing the participant's legs in hot water (43 °C). During the heating period, the participants performed 1.5 minutes of isometric hand-grip exercise at 40 % of maximum voluntary contraction with or without (Control) post exercise occlusion of the limb with a pressure cuff to stimulate muscle metaboreceptors for 9 minutes. *Study ΙΙ*: Twelve (6 for each sex) young subjects performed the similar forearm muscle metaboreceptors stimulation while cycling for 13.5 minutes at the exercise intensity of 40 % maximum oxygen consumption while wearing the water perfused suit.

## Results

S*tudy Ι*: Forearm muscle metaboreceptors stimulation significantly lowered Δmean body temperature thresholds for sweating and cutaneous vasodilation on the oppositional arm of hand-grip exercise during passive heating compared with Control (*P *<0.05) without differences in thermosensitivity of the responses. *Study ΙΙ*: Forearm muscle metaboreceptors stimulation did not significantly (*P *> 0.05) affect the onset threshold and thermosensitivity for sweating and cutaneous vasodilation during exercise.

## Discussion

Our results suggest that forearm muscle metaboreceptors activation can modulate heat loss responses during a passive heat stress only. The acceleration of sweating and cutaneous vasodilation through a reduction in the core temperature thresholds of the responses would suggest a central modulation of temperature regulation [2]. It is thought that any of overriding factors associated with dynamic exercise may be masking the influence of forearm muscle metaboreceptors activation.

## Conclusion

Forearm muscle metaboreceptors activation lowers core temperature thresholds for heat loss responses during passive heating but not during exercise.

## References

[B1] Kondo N Nishiyasu T Inoue Y Koga S Non-thermal modification of heat-loss responses during exercise in humans Eur J Appl Physiol 2010 110 447 458 10.1007/s00421-010-1511-x 20512585

[B2] Nadel ER Mitchell JW Saltin B Stolwijk JA Peripheral modifications to the central drive for sweating J Appl Physiol 1971 31 828 833 5123659 10.1152/jappl.1971.31.6.828

